# Mitochondrial oxidative stress in the tumor microenvironment and cancer immunoescape: foe or friend?

**DOI:** 10.1186/s12929-022-00859-2

**Published:** 2022-09-26

**Authors:** Cheng-Liang Kuo, Ananth Ponneri Babuharisankar, Ying-Chen Lin, Hui-Wen Lien, Yu Kang Lo, Han-Yu Chou, Vidhya Tangeda, Li-Chun Cheng, An Ning Cheng, Alan Yueh-Luen Lee

**Affiliations:** 1 National Institute of Cancer Research, National Health Research Institutes, 35 Keyan Road, Zhunan, Miaoli 35053 Taiwan; 2 Joint PhD Program in Molecular Medicine, NHRI & NCU, Zhunan, Miaoli 35053 Taiwan; 3 Liver Research Center, Linkou Chang Gung Memorial Hospital, Taoyuan, 333 Taiwan; 4 Genomics Research Center, Academia Sinica, Taipei, 115 Taiwan; 5 Department of Life Sciences, College of Health Sciences and Technology, National Central University, Zhongli, Taoyuan 32001 Taiwan; 6 Graduate Institute of Biomedical Sciences, China Medical University, Taichung, 40402 Taiwan; 7 Department of Biotechnology, College of Life Science, Kaohsiung Medical University, Kaohsiung, 80708 Taiwan

**Keywords:** Tumor microenvironment, Mitochondrial reactive oxygen species (mtROS), Inflammation, Hypoxia, Immunoescape, Combination cancer immunotherapy, Mitochondrial chaperone, Lon protease (LonP1), Cisplatin resistance

## Abstract

The major concept of "oxidative stress" is an excess elevated level of reactive oxygen species (ROS) which are generated from vigorous metabolism and consumption of oxygen. The precise harmonization of oxidative stresses between mitochondria and other organelles in the cell is absolutely vital to cell survival. Under oxidative stress, ROS produced from mitochondria and are the major mediator for tumorigenesis in different aspects, such as proliferation, migration/invasion, angiogenesis, inflammation, and immunoescape to allow cancer cells to adapt to the rigorous environment. Accordingly, the dynamic balance of oxidative stresses not only orchestrate complex cell signaling events in cancer cells but also affect other components in the tumor microenvironment (TME). Immune cells, such as M2 macrophages, dendritic cells, and T cells are the major components of the immunosuppressive TME from the ROS-induced inflammation. Based on this notion, numerous strategies to mitigate oxidative stresses in tumors have been tested for cancer prevention or therapies; however, these manipulations are devised from different sources and mechanisms without established effectiveness. Herein, we integrate current progress regarding the impact of mitochondrial ROS in the TME, not only in cancer cells but also in immune cells, and discuss the combination of emerging ROS-modulating strategies with immunotherapies to achieve antitumor effects.

## Background

Reactive oxygen species (ROS) are a group of highly reactive oxygen-containing molecules generated through several mechanisms in cells, such as aerobic respiration in mitochondria, metabolic enzymes, peroxisomes, and membrane-bound NADPH oxidases (NOXs) [1, 2]. Although generated via several sources, mitochondria are the major cellular organelles of ROS production; mitochondrial ROS (mtROS) are mainly produced by the electron transport chain (ETC) and oxidative phosphorylation (OXPHOS) during aerobic respiration. The superoxide (O_2_^–^), for example, is produced from incomplete electron transfer and leakage of electrons through ETC Complexes I, III, and IV [3, 4]. Due to the multifaceted role of ROS in cell survival and function, intracellular ROS levels must be strictly controlled to maintain the equilibrium between ROS production and scavenging through multiple mechanisms. At high levels, ROS cause oxidative damage to DNA, proteins, and lipids, and become deleterious to cells. At low to medium levels, ROS also act as a cellular signaling messenger, involved in regulating several varieties of cellular functions including gene expression, proliferation, differentiation, and stress response. In other words, the imbalance of ROS level decides the severity of the oxidative stress for either the cellular compromised or survival-associated functions. Simultaneously, ROS can be regulated and controlled by their localization within the cell, i.e., cells have several protective mechanisms against ROS via compartmentalization [5]. In cancer cells, mitochondria can also be redistributed to these regions of the cell to provide energy demands for cell migration under oxidative stress [6]. Further mechanisms to localize ROS and allow for a restricted response include the control of mitochondria turnover and localization. For example, mtROS can be eliminated by mitophagy that removes damaged ROS-producing mitochondria through targeted autophagy [7]. In addition, uncoupling of OXPHOS is involved in the control of mtROS production. During aerobic respiration, mitochondrial uncoupling has been considered as a cytoprotective strategy under oxidative stress, including inflammation, aging, diabetes, or atherosclerosis [8–10]. However, mitochondrial uncoupling proteins (UCPs) lower the efficiency of OXPHOS and are involved in the increase of mtROS production in cancer [11]. Mitochondrial calcium ([Ca^2+^]_m_) is another factor to upregulate the entire OXPHOS machinery, resulting in faster respiratory chain activity. [Ca^2+^]_m_ coupled with proton uncouplers showed significance in promoting mtROS [12, 13]. Likewise, mtROS generated by the OXPHOS uncoupler CCCP (Carbonyl cyanide m-chlorophenyl hydrazone) is important for the Peroxiredoxin 6-induced PINK/Parkin mitophagy [14].

The imbalance of redox homeostasis is detrimental to biomolecules, cells, and even entire organism. It has been well known that cancer cells carry more ROS than their normal surrounding cells. Many pro-tumor events promote ROS production, including activation of oncogenes, loss of tumor suppressor function, changes in mitochondrial activity, adaptation to hypoxia, altered stromal interactions, fibrosis, and pathophysiology of inflammation. Some researchers have shown that superoxide-dependent oxidative stress may be involved in the pathophysiology of inflammation, fibrosis, and cancer [15, 16]. For instance, it is well demonstrated that ROS activate mitogen-activated protein kinase (MAPK) family comprising of JNK, p38, and ERK [17]. These MAPK family members function in a cellular context-specific manner, integrating signals that regulate proliferation, survival, apoptosis, and invasiveness [18, 19]. However, the consequences of ROS are very different, and ROS act as a double-edged sword in carcinogenesis, which both support and inhibit malignant behavior, a foe and friend [20–22]. The biological function and the therapeutic strategies of oxidative stress in cancer biology have been comprehensively described in other reviews [20, 23–25]. Under sustained ROS stress, it will potentially cause serious damage to cell structure and function, which also induces somatic mutation [26]. For example, ROS can damage both nuclear DNA (nDNA) and mitochondrial DNA (mtDNA), which leads to mutagenesis and elicits the metabolic reprogramming causing an increasing risk of carcinogenesis [27, 28]. mtROS damage mtDNA and causes adaptation of metabolic reprogramming, which are required for tumorigenesis. Therefore, to protect against ROS, cells develop antioxidant defense mechanisms for their elimination, which include endogenous and exogenous as well as enzymatic and non-enzymatic antioxidants. The superoxide dismutase (SOD) is the first antioxidant enzyme characterized, which can dismutate two superoxides (O_2_•^–^) into H_2_O_2_ and O_2_ [29, 30]. Catalase is then responsible for detoxifying the H_2_O_2_ into water. Glutathione (GSH), is another endogenous antioxidant mechanism within the cells [31]. Glutathione peroxidase (GPx) is a group of enzymes capable of reducing hydroperoxides using GSH as a substrate [32]. Regarding non-enzymatic mechanisms, mitophagy is an important form of autophagy for the selective removal of dysfunctional mitochondria and the elimination of mtROS [33]. These mtROS produced by dysfunctional mitochondria also can promote tumor development, possibly by perturbing the signal transduction adapter function of p62-controlled pathways [34]. Ironically, antioxidant defense mechanisms are also considered to show that control of increased ROS, which could promote tumorigenicity.

Since ROS can damage both nDNA and mtDNA, deregulated high ROS production in cancer cells may occur due to exogenous chemotherapy and radiotherapy (RT). Explicit role of high ROS level in cellular-intrinsic events of cancer leads to cell death and benefits the treatments of chemotherapy and RT. The elevated ROS levels in cancer have been shown to induce tumor cell death and increase sensitivity to anti-tumor therapy. In addition, growing evidences suggest that eliminating damaged mitochondria by selective autophagy is a powerful tool to control the inflammation in the immune system [35]. Therefore, the demand for the understanding of the complexity of ROS in malignancies will be key to exploring the potential of ROS-targeting therapies for cancer.

Recently, cancer biology is evolving from a 'cancer cell-centric' perspective to a systematical concept that considers cancer cells as a network of surrounding cells, which is called a tumor microenvironment (TME) [36]. The TME mainly includes tumor cells and their neighbor cells, including cancer‑associated fibroblasts (CAFs), vascular endothelial cells, and immune cells. By interacting with these neighbor stromal cells through soluble factors and signaling molecules, tumor cells have developed adaptive mechanisms to survive under various extreme conditions of the TME, such as hypoxia, higher ROS, and lower pH [37–39]. These stress phenotypes are common characteristics of many tumor types and so called the hallmarks of cancer [37, 40]. According to the hypothesis of ‘seed and soil’ first proposed by Paget in 1989, where tumor cells were known as ‘seeds’ and the surrounding microenvironment was known as ‘soil’ [41]. To survive under these environmental stresses, cancer cells in the TME activate the stress response, such as escape in apoptosis, angiogenesis that supplies their need for oxygen and nutrients, immunosuppression, invasion, and metastasis. ROS are associated with inflammation and cancer development as well as progression. This persistent inflammatory/oxidative environment leads to a vicious cycle that damages healthy adjacent epithelial and stromal cells, ultimately leading to carcinogenesis [42–44]. Furthermore, it is important to note that the TME significantly contributes to cancer development through creating an immunosuppressive environment that ultimately causes the suppression of cytotoxic T lymphocytes (CTL) response [45]. Similarly, ROS act as a double-edged sword and play a dual role in immune responses. One of ROS role in anti-cancer function is through the activation of T cells and NK cells to increase the ROS production, which allows the neutrophils and macrophages recruitment to kill cancer cells [46]. On the other hand, the elevated ROS can support cancer cells through promoting tumor-contributing immune cells, including myeloid-derived suppressor cells (MDSCs), tumor-associated macrophages (TAMs), and regulatory T cells (Tregs). In conclusion, the production and regulation of ROS levels in the TME-associated cancer and stromal cells play a decisive role in the progression of the disease. mtROS function is tightly controlled to maintain the balance through multiple mechanisms which are involved in inflammation and tumorigenesis [47]. However, extensive research is necessary to unveil the critical regulatory mechanisms driven by mtROS in tumor and tumor infiltrating immune cells for immune response in the TME. In this review, we will interpret how tumor cells process the mitochondrial ROS regulation to interact the components in the TME by different mechanisms and aspects: (1) The impact of mitochondrial ROS on the survival signaling in cancer cells; (2) The impact of mitochondrial ROS on inflammation and cancer immunoescape in the tumor microenvironment (TME); (3) The impact of mitochondrial ROS on immune cells in the TME; (4) The translational significance of mitochondrial ROS modulation in the prognosis and combination of cancer immunotherapy.

## The survival signaling of mitochondrial ROS stress by chaperone in cancer cells

**Fig. 1:**
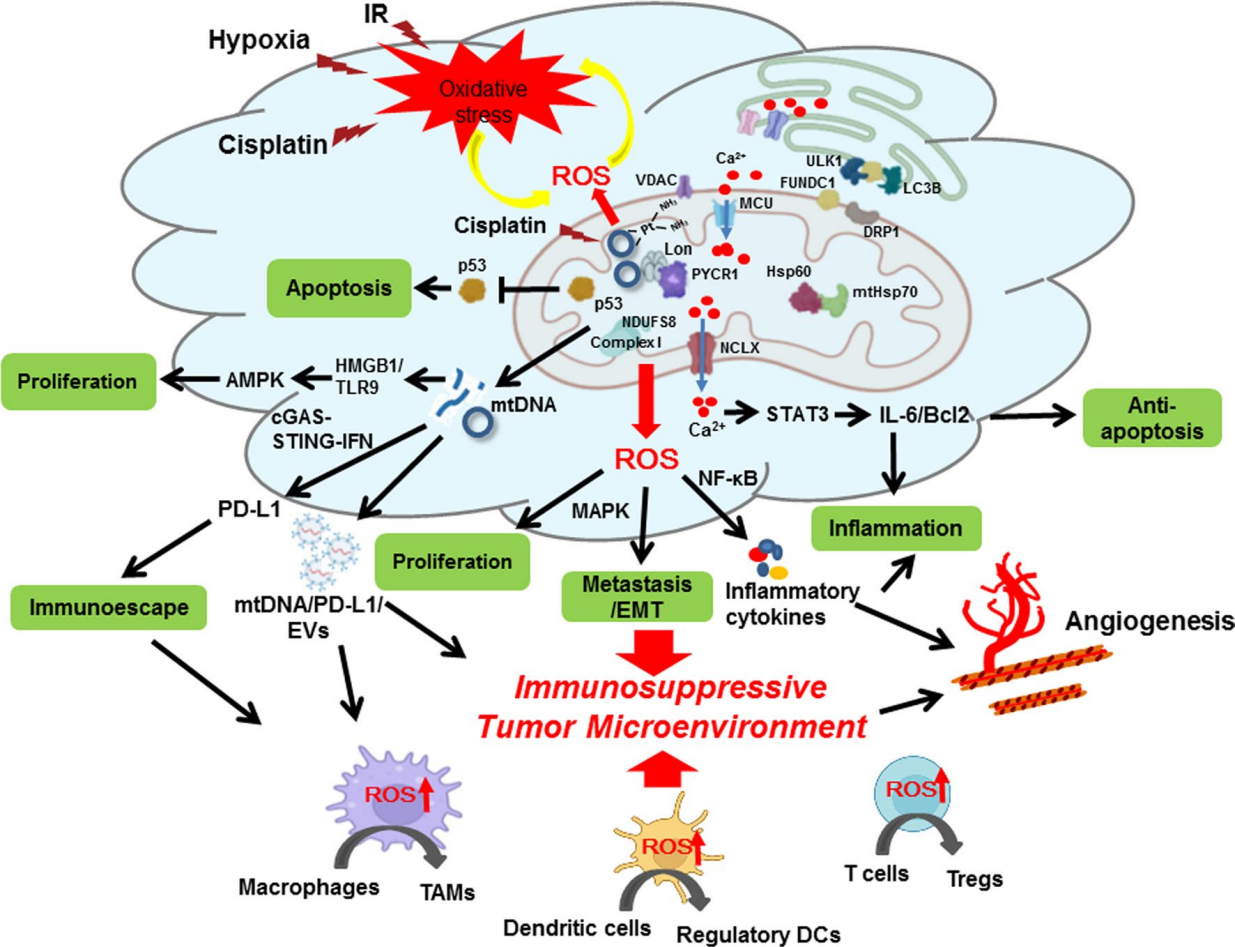
Scheme of mitochondrial ROS stress promotes cell survival and inflammation that causes an immunosuppressive tumor microenvironment (TME) to induce tumorigenesis. Mitochondria are the major cellular source of ROS generation. Mitochondrial ROS (mtROS) are mainly produced by mitochondrial aerobic respiration or as a byproduct of the activity of metabolic enzymes. Chaperone Lon is the major one of mitochondrial protein quality control system. Lon binds with NDFUS8 in the Complex I of electron transport chain and with PYCR1 reductase to up-regulate mtROS generation to promote cell proliferation and inflammation. Mitochondrial chaperone complex of HSP60-mtHSP70-Lon sequesters p53 in mitochondria matrix and stabilizes with NCLX (Na^+^/ Ca^2+^ exchanger) to restrain apoptosis and increase the cisplatin resistance under ROS stress. In addition, mtROS cause the oxidative damage on mtDNA and induce IFN signaling that upregulates PD-L1 expression to inhibit T-cell activation. Under ROS stress, cancer cells to secrete NF-κB-dependent inflammatory cytokines ( IL-6, IFN-γ, TGFβ, VEGF, IL-4, and IL-10) to cause the immunosuppressive state of macrophages, dendritic cells (DC), and T cells (Treg). Upregulation of Lon by ROS and hypoxia also induces the secretion of extracellular vehicles (EVs) that carry mtDNA and PD-L1. mtROS-induced EVs further induce the production of IFN and IL-6 from macrophages, which attenuates T-cell immunity in the TME. Macrophage-induced ROS leads to the accumulation of Treg and regDC cells. In short, mtROS cause an immunosuppressive TME to promote immunoescape, survival, and EMT/metastasis of cancer cells

Intracellular ROS mainly come from dysfunctional mitochondrial respiratory chain enzyme complexes [3, 4, 48] and are crucial intermediates to trigger cellular signaling promoting and suppressing tumorigenesis [17, 21, 49, 50]. Mitochondria contribute to cellular energy metabolism, redox status, calcium homeostasis, and cell death regulation in mammalian cells. Therefore, mitochondria are the sensors of environmental stresses and responders to various stresses by regulating a series of signals to communicate with the other organelles to reduce the impact of subsequent stress damages. Several factors, such as mtDNA metabolism/damage, metabolic enzyme defects, and morphology dynamic changes, contribute to mitochondrial dysfunction in cancer cells under severe stress phenotypes. Furthermore, as the center of energy metabolism and programmed cell death, the precise harmonization between mitochondria and other organelles in the cell is absolutely vital to the survival of cancer cells [51]. Here, we specifically focus on the survival strategies in cancer cells for the oxidative stress by mitochondria (Table 1 and Fig. 1).

**Table 1:** The underlying mechanisms and dual roles of mitochondrial ROS stress in the tumor microenvironment

Cancer cells	Molecular/signaling events of ROS induction	Foe	Friend	Cellular/system events	References
	MAPK cascades/ ERK1/2/ JNKs/p38	√		Proliferation, survival, migration	[17–19, 81]
	Mn-SOD / antioxidant	√		Antioxidative system	[29, 30, 112, 113]
	GSH/ antioxidant	√		Antioxidative system	[31, 32]
	NF-κB	√		Cytokines/Inflammatory response	[81]
	TGF-β	√		Tumorigenesis/ Inflammatory response	[81, 87–94]
	Mitophagy/NIX/BNIP3	√		Cell survival/dysfunctional mitochondria removal/ anti-apoptosis	[261, 292–295]
	Mitophagy/PINK-Parkin	√		Cell survival	[120, 121]
	Hsp60 /mtHsp70/Mitochondrial chaperones	√		Protein refolding/UPRmt Proliferation and metastasis	[57–62, 82]
	Lon/Mitochondrial chaperones	√		Elevated mtROS/ tumorigenesis	[48, 79–85]
	Intracellular Ca^2+^ increasing under hypoxia	√		ROS generation /tumorigenesis/ drug resistance	[84, 95, 101–106, 108, 296]
	Increasing Ca^2+^ in mitochondria		√	Cytochrome C releasing/apoptosis	[95, 96, 109]
	p53		√	Apoptosis	[83, 293]
	Autophagy	√	√	ROS suppression/ Cell survival /Cell death	[35]
	PTEN		√	Neutralizes intracellular ROS/immune escape	[297]
	PD-L1	√		Immune resistance	[131, 141, 219]
	STAT3	√		Inflammatory response/IL-6	[84, 138]
	HIF-1	√		ROS generation/ Cell survival/ Tumorigenesis	[121, 123, 150–152]
	mtDNA oxidation/damage	√	√	ROS generation/Cell death/ Inflammation/ Drug resistance	[131, 156–159, 212–214, 298, 299]
	Cisplatin-induced mtDNA damage		√	ROS generation/ Cell death/ Ca^2+^-dependent cisplatin resistance	[84, 96, 156, 222, 263]
Immune cells	PD-1/PD-L1	√		Immune inhibition	[141]
	CD39/CD73	√		Immune evasion of T cell	[144, 145]
	TCR/CD8-MHC	√		Immune suppression of T cell	[146]
	Arginase-1/nitric oxide /peroxynitrite	√		Inhibit T cells proliferation	[147–149]
	Fas	√		T cell apoptosis	[164]
	IL-15		√	NK cells resistance against oxidative stress	[163]
	DAMPs/HMGB1		√	DC activation and ultimately antitumor T cell responses	[166–168]
	NOX2		√	Myeloid cell ROS production	[169]
	Thioredoxin-1	√		Antioxidative function for regulatory T cells	[171, 172]
	Glutathione	√		Restricting serine metabolism to preserve Treg function	[173]
	M1 macrophage signaling pathway		√	Tumor suppression	[179, 180, 199]
	M2 macrophage polarization	√		Promote tumor progressions	[81, 182, 183, 199, 300]
	IL-6	√		Tumor-associated macrophage differentiation [TAM]	[185, 186]
	Proinflammatory cytokines, or TLR ligands, LPS, IL-6		√	Mature DCs drive effector T cell response	[190]
	IL-10, TGF-β, Vit-D3	√		Regulatory DCs [regDCs] dampen effector T cell differentiation or activate Tregs	[190]
	Oxidatively truncated lipid bodies in DCs		√	Deaden CD8 T cells responses	[301]
	Ebselen	√		Inhibits ROS production, DC-T cell cross talk for cytokine production	[204]
	mtROS elevation	√		Obstructed antigen presentation disrupt DCs/T cell	[205]
	ROS-triggered ER stress	√		Inhibition of IL-1β, CD86, and IL-12 in DCs to inhibit effective T cells	[302]

### Mitochondrial protein quality control in cancer cell survival

Mitochondrial protein homeostasis or protein quality control (mtPQC) is dependent on the normal function of protease and chaperone system [52]. The mtPQC system is essential for maintenance of proteostasis in mitochondria by trying to refold or by degradation of damaged proteins. Typically, the degradation of misfolded proteins is performed by ATP-dependent proteases of the AAA + (ATPases associated with a wide variety of cellular functions) family. Mitochondria contain several different AAA + proteases, LonP1, CLPP (CLP protease proteolytic subunit), and YME1L1 (ATP-dependent zinc metalloprotease) [53, 54]. The mitochondrial stress through deregulation in proteostasis generates internal imbalance which leads to mitochondrial unfolded protein response (UPRmt). UPRmt can be due to the elevated mtROS, decrease in mtDNA number or mitochondrial mass, impairment of the protein quality control system and disorder caused by oxidative phosphorylation [55, 56]. Hence, the cell activates the adaptive transcriptional regulatory response to promote the cell survival through recovery of mitochondrial function, adapting metabolism and the innate immunity. Accumulating evidence has reported that HSP60 and mtHSP70 play a chaperone role in cancer proliferation and metastasis through maintain a quality genome and assisting for refolding of unfolded and misfolded proteins [57]. The transcription factor activating transcription factor 5 (ATF5) regulates the gene expression of HSP60, GRP75 (mtHSP70) and other proteases for cancer cell survival and resistance against therapeutics and apoptosis [58, 59]. HSP60 plays an inhibitory role against cell death through its interaction with surviving and cyclophilin-D [60, 61]. Similarly, during hypoxia, mtHSP70 translocation to the outer membrane of mitochondria to interact with hypoxia-inducible factor 1 (HIF-1) leading to truncation of VDAC and thereby developing chemotherapeutic resistance by inhibiting apoptosis [62]. Also, mtHSP70 interaction with podoplanin (PDPN) regulates the growth and invasion of oral squamous cell carcinoma [63]. UPRmt-activated AAA proteases such as Lon and ClpP are responsible for maintaining mitochondrial homeostasis by removing harmful proteins [64]. Lon protease (LonP1) is a highly conserved, main, and abundant proteases located in the mitochondrial matrix. Mitochondrial Lon is a multi-functions protease as well as a stress protein which is induced by multiple stresses, such as starvation, ER, hypoxia, oxidative stress [65]. Elevated mtROS in depolarized mitochondria was suppressed through Lon-ClpP proteolytic quality control axis by degrading the Complex I ROS-generating domain [66]. Lon protease activity was increased in higher folds upon AKT phosphorylation of Lon. In addition, Lon interaction with FUN14 domain-containing protein 1 (FUNDC1) protects cancer cells from ROS accumulation through stabilizing ETC Complex II and Complex V [67, 68]. Lon capacity in proteostatic stress response to degrade the unfolded cytosolic proteins imported after mitochondrial FUNDC1 and cytosolic HSC70 interaction [69]. The common substrates of Lon and ClpP are involved in the regulation of metabolic functions including amino acid, oxidative phosphorylation (OXPHOS), and lipid metabolism [70].

Given that these stresses are commonly happened in various cancers, it is nothing remarkable that Lon is upregulated in fast-growing tumors and needed for cancer survival. Indeed, mitochondrial Lon protease upregulation has been found in many different human cancers, including non-small cell lung cancer [48, 71], malignant B cell lymphoma [72, 73], cervical cancer [74], bladder cancer [75], prostate cancer [76], colon cancer [77, 78], and oral squamous cell carcinoma cell lines [48]. Increasing evidence supports that downregulation of Lon impairs the structure and function of mitochondria to cause cell death [79, 80]. Mitochondrial Lon regulates the Complex I of electron transport chain and PYCR1 to up-regulate ROS generation to promote cell proliferation and transformation [48, 81]. As a cytoprotective chaperone, Lon interacts with Hsp60-mtHsp70 complex [82] and sequesters p53 [83] in mitochondria matrix to restrain apoptosis. A recent study also showed that the resistance mechanism by Lon interacting with NCLX inhibits excess mitochondrial calcium influx induced by cisplatin to trigger cell death [84]. Lon also participates in cysteine metabolism to repress lipid peroxidative in regulating ferroptosis [85]. In summary, these studies indicate that mitochondrial chaperone as a key factor to maintain sustaining proliferative signaling and resisting cell death in cancer cells (Fig. 1).

### Mitochondrial ROS stress and epithelial mesenchymal transition (EMT) and metastasis

Several studies disclosed the chaperone activity of mitochondrial Lon and showed upregulation of Lon induced ROS generation playing a role in stress signaling [48, 81–83]. Lon can cooperate with other mitochondrial proteins such as NDFUS8 [48] or PYCR1 [81] to induce mitochondrial ROS generation; on the contrary, downregulation of Lon reduced mitochondrial ROS production [75]. High expression of Lon promotes the progression of tumorigenesis, such as metastasis and invasion were found in both cancer cell models [48, 78, 81, 86] and nude mice [77, 81]. In cancer cells, several signaling activation involved in tumorigenesis were referred to under control of Lon-induced ROS. For example, Ras-ERK(ERK1/2), MAPK(P38) [48, 81], and WNT (β-catenin) [78] signaling activation increased cell proliferation. Cell migration and invasion are cancer survival strategies to escape from lots of stresses in the TME. Increasing cell migration ability induced by EMT processes was through Lon-induced ROS MAPK or NF-κB pathways [48, 81].

Among cytokines releasing from Lon overexpression cancer cells, TGF-β upregulation appeared both in cancer cells and the microenvironment [81]. In most solid tumors, unlike early-stage cancers, TGF-β signaling promote a range of tumor-promoting effects. Even in cutaneous T cell lymphoma (CTCL), TGF-β mediated cell migration is regulated by NF-kB [87]. Many papers have proposed that TGF-β1 whereby different mechanisms stimulate mtROS production. [88–92]. Ishikawa et al. further showed that TGF-β1 induced mtROS production and underlay the activation of genes associated with EMT [89]. Previous studies also pointed out that Lon-induced ROS upregulates TGF-β through p38-NF-κB signaling [81, 93, 94], suggesting mitochondrial Lon contributes to the immune-suppressive microenvironment required TGF-β-mediated EMT and inflammatory response. In summary, the mitochondrial Lon in the matrix can interact with different proteins in the mitochondria under different stresses to regulate ROS generation and further activate downstream ROS-mediated signaling pathways to promote tumorigenesis and metastasis.

### The interplay between calcium and ROS in cancer cell survival

Many physiological and pathophysiological processes were associated with calcium (Ca^2+^) and ROS, resourceful signaling molecules, and their mutual interplay can regulate the dysfunctional mitochondria and maintain mitochondrial bioenergetics. The relationship between calcium and ROS is mutual. Calcium is a secondary messenger that controls various cellular functions from cell signaling, secretion, metastasis, autophagy, and cell death, and Ca^2+^ also communicates with other systems particularly ROS [95]. Calcium regulates oxidative phosphorylation by activating enzymes isocitrate dehydrogenases, pyruvate dehydrogenases, α-ketoglutarate dehydrogenases, and ATP synthesis in mitochondria, which increases metabolic rate and thereby leaks respiratory chain electrons producing mitochondrial ROS [12, 95]. Studies on ovarian cancer denoted that intracellular ROS levels were modulated by calcium in cytosol and mitochondria under cisplatin treatment in cisplatin sensitive and resistant SKOV3 cells. Treatment of BAPTA-AM (a Ca^2+^ chelator) or 2-APB (an IP3R inhibitor) decreased intracellular ROS in SKOV3 cisplatin sensitive cells and protected cancer cells from apoptosis. Therefore ROS and Ca^2+^ mutual interplay in chemotherapy decide the fate of cells [96]. Calcium channels like voltage dependent Ca^2+^ channels (VDCC), Store operated channels, TRP channels, and IP_3_R are redox regulated because of the presence of cysteine residue in their domains [95, 97–100]. Takahashi et al., found that ROS-activated TRPA1 calcium channel increased intracellular calcium and activated calcium mediated pro-survival pathways PI3K/AKT, mTOR, RAS-ERK [101, 102].

Mitochondrial Ca^2+^ uptake influences cellular Ca^2+^ signals to generate ATP synthesis through Complex V, the F0F1 ATPase activity. Mitochondrial calcium uniporter (MCU) is the selective channel responsible for mitochondrial Ca^2+^ uptake leading to mtROS generation and HIF1α signaling events for breast cancer progression [103, 104]. The mtROS and total ROS generated after MCU mediated calcium uptake leading to trigger signaling events by inhibiting the NAD + /SIRT3/SOD2 pathway [105]. Under hypoxia-induced oxidative stress, mtROS generation upon mtCa^2+^ uptake is dependent on S-glutathionylation of MCU cysteine 97 (Cys-97) residues without any involvement of MCU regulators [106]. Like MCU, the efflux channel of mitochondria also plays a role in generating mtROS for cellular activity under hypoxia. Acute hypoxia causes the activation of NCLX through Complex I inactivation and allows the mitochondrial Na^+^ import/ mtCa^2+^ export to cytosol. This leads to a consequence to increases superoxide production at Complex III, generating a redox signal [107]. Impact of acute hypoxia-induced mitochondrial ROS activates STIM1 puncta formation and SOCE activation through HIF1α and subsequent Ca^2+^ signal benefits tumor cell proliferation [108].

The physical interactions between ER and mitochondria called as mitochondria associated membranes (MAMs) are hotspots of calcium regulation, which accumulate calcium into mitochondria in chemotherapy leading to cancer cell death [109]. Redox nanodomains at ER-mitochondria contact sites increase calcium influx into mitochondria and regulate calcium oscillations by IP3R channels and metabolic activities of cells [110]. ER-mitochondria contact (MAM) are enriched with the proteins responsible for Ca^2+^ and ROS transport between mitochondria and ER. MAMs contains ER-localized IP3R/RyR receptors, SERCA pumps, mitochondrial Voltage-dependent anion channel (VDAC), and the mitochondrial Ca^2+^ uniporter (MCU) in the outer and inner mitochondrial membrane [111]. To avoid the lethal cell death triggered by mtROS, cells set up the preventive mechanism to neutralize the mitochondria generated ROS through activation of mitophagy, release of mitochondrial calcium through NCLX efflux channel [84], MnSOD enzymatic dismutation [112, 113]. Taken together, it is important that, for the normal function of mitochondria Ca^2+^ and ROS homeostasis are very signification, a little dynamic alterations or imbalance in calcium leads to different consequences on cellular function especially during pathophysiological process. Thus understanding these interconnecting molecules related pathways would pave for discovery of novel drug therapies in diseases.

### Mitochondrial ROS-induced mitophagy under hypoxic resistance

The mitophagy activation and sustainability are important for the cancer cells to develop hypoxic resistance property among other alternative pathways is depending on the severity and duration of hypoxia. This balancing act during hypoxia is strongly dependent on HIF1α, mTOR and UPR [114]. Hypoxia drives the receptor mediated mitophagy through participation of several protein components in conjunction with key regulatory receptor molecule like Bnip3-like/NIP3-like protein X (BNIP3L/NIX), Bcl-2/Adenovirus E1B 19 kDa-interacting protein 3 (BNIP3), and FUNDC1 [115–117]. mtROS plays a very prominent role during hypoxia for the activation of mitophagy receptor, Recently, in glioblastoma cells, hypoxia induced NIX promotes mitophagy and regulates mtROS on associating with GTPase RHEB (controls OXPHOS activity), and further regulating the mTOR/AKT/ HIF1α signaling axis [118, 119]. Contrarily, it is also suggested that hypoxic cell survival majorly relies on receptor mediated mitophagy/independent of PINK-Parkin mediated mitophagy in cancer cells depending on the behaviour of mitochondria during hypoxia. In argument to this, HEY1 and PINK1 expressions were reciprocal to each other and has shown poor clinical outcomes. HIF1/HEY axis overcomes oxidative stress through repressing PINK1 which is responsible for mitochondrial biogenesis and suppresses ROS level to make mitochondria less reliable for HCC survival [120, 121]. FUNDC1, a hypoxia specific mitophagy receptor, conducts mitophagy independent of PINK/parkin function and regulates mitochondrial homeostasis. Mitochondrial dysfunction under hypoxia allows increase in MAM formation and FUNDC1 also reported to regulate mitochondrial dynamics at the MAM region by regulating the dynamic related proteins DNM1L/Drp1 and OPA1 [122]. Recently, FUNDC1 dependent MAM formation promotes the angiogenesis in endothelial cells [123]. The hypoxia dependent receptor mediated mitophagy activation is completely dependent on the tumor heterogeneity and the resistance development property by mitophagy is dependent on the severity of mitochondrial damage pattern caused in hypoxic TME.

## The impact of mitochondrial ROS stress on inflammation and cancer immunoescape in the tumor microenvironment (TME)

To survive under the ROS stress in the TME, cancer cells activate the stress response of escape in apoptosis, metastasis, and immunosuppression/immunoevasion. Cancer immunoevasion is still a great barrier in the current immunotherapy treatment. The host tumor response in context to control tumor development and progression is through the immunoediting process which are majorly staged into three steps (1) elimination, (2) equilibrium, and (3) escape [124, 125]. The cancer immunoescape gains advantage in immunosuppressive TME through developing abnormalities in antigen presenting and anti-tumor cells (T cells, Dendritic cells), developing immune resistant tumor cells or posting a blockade during T cells trafficking [126]. Emerging evidence showed that ROS are not only mediators of oxidative stress but also players of immune regulation in tumor development [127]. In this review, we will discuss how ROS and hypoxia modulate tumor and immune cells in the TME, which regulates inflammation and causes immunoescape (Table 1 and Fig. 1).

### Mitochondrial ROS stress in the TME

It is generally accepted that the TME is a chronic inflammatory environment that contributes the development and progression of most tumors. There is growing evidence that the mitochondrial ROS play a "central" role in inflammatory TME that ultimately exacerbates cancer [47, 128]. Within the TME, active oncogenic signaling promotes cancer cells to autocrine and paracrine small molecular or cytokines to surrounding cells for tumor promotion. In cancer cells, elevated ROS have been shown to contribute to metastasis and angiogenesis through the secretion of inflammatory cytokines, the stabilization of HIF, and activation of AMPK signaling networks to enhance NADPH production. The ROS property in TME has been implicated with immune cell activation and suppression determining the cancer status. TME influences the PGC1α expression, an important contributor in mitochondrial biogenesis, to promote the accumulation of tumor-infiltrating T cells to resume anti-cancer activity. On the other hand, high ROS inhibits T cell responses by suppressing the formation of TCR and MHC antigen complex, which promotes cancer progression through evading immune response [129]. Previously, the mechanisms of how oxidative stress modulates chronic inflammation-induced carcinogenesis from the TME point of view described in other reviews [5, 47, 130]. In this review, we mainly discuss the impact of mtROS stress on the TME, including cancer cells and various types of immune myeloid cells (Fig. 1).

The cancer cells attempt to evade the anti-tumor surveillance system which are termed as adaptive immune resistance. To avoid immune destruction, tumor cells hijack the physiological immune response caused by the activated T cells. mtROS are used by cancer cells and immunosuppressive immune cells to create immune tolerance to tumors [69–76]. The mitochondrial Lon has been shown to regulate the balance of mtROS production through cooperation with different proteins in the mitochondria [48, 66, 81–83, 131, 132]. Moreover, the mitochondrial Lon-induced mtROS-NF-κB axis stimulates inflammatory cytokines releasing from cancer cells to establish immune suppression of the TME [81]. Interestingly, mitochondrial Lon promotes tumorigenesis in an NF-κB-dependent manner, but Lon expression is also repressed by IκB kinase (IKK) inhibitor VII (IKKi7) [81]. Among ROS-induced inflammatory signaling, NF-κB is constitutive activation in many different types of cancer and promotes a variety of inflammatory factors [133, 134]. Another well-known signaling that responds to ROS induction in mediating tumorigenesis is MAPK cascades [135]. ROS-induced MAPK activation can also regulate NF-κB signaling to promote inflammatory factors secretion, such as IL-1β, IL-6, and TNFα [133, 136, 137]. Kuo et al., also especially pointed out that NF-κB and MAPK promoting inflammatory cytokines section, such as IL-6 and VEGF are regulated by mitochondrial Lon-induced ROS [81]. Another hypoxic factor, HIF1α can assist a series of kinase cascades activation leading to STAT3 to promote IL-6 secretion [138]. In addition, HIF1α is a key stimulator to induce upregulation of mitochondrial Lon that generates ROS [48]. A recent study also indicated that under cisplatin treatment, IL-6 secretion promoted by STAT3 signaling is dependent on Lon-induced increase in intracellular ROS and calcium [84]. Therefore, the positive feedback of Lon-induced ROS via NF-kB axis enhanced downstream signaling to promote tumor progression.

ROS exert a significant impact on the expression of programmed cell death protein 1 and programmed cell death-ligand 1 (PD-1 and PD-L1). The variable PD-L1 response to ROS modulation reflects the complexity of ROS biology in the TME. Through binding with PD-1, an inhibitory immune checkpoint receptor expressed on activated immune cells, PD-L1 (B7-H1) on tumor cells attenuates the effector function in dendritic cells [139] and macrophages [140]. In addition, HIF-1α contribution to positive PD-L1 expression is ROS dependent and this accompanies the infiltration of tumor supportive immune cells, myeloid-derived suppressor cells (MDSCs), regulatory T cells (Treg), and tumor-associated macrophages (TAMs) [141]. TAMs integrate the multiple molecular links between ROS and PD-L1. Elimination of ROS through redox-active drug MnTE-2-PyP5 + selectively inhibits M2 macrophage polarization and pro-tumorigenic function [142, 143]. Treg cell apoptosis triggered by oxidative stress is a novel tumor immune-evasion mechanism in the TME. Apoptotic Treg cells efficiently convert ATP into immunosuppressive adenosine via CD39 and CD73 in vitro and in vivo [144, 145]. MDSCs often represent the major producer of oxidizing species in the TME which undergoes massive expansion during tumor progression. ROS through MDSCs shows immune suppression capacity through modifying TCR and CD8 channels, leading to the antigen specific tolerance of peripheral CD8 + T cells upon CD8 + T cells losing their ability to bind phosphorylated MHC [146]. MDSCs suppress T cell proliferation also through increasing the production of Arginase-1, nitric oxide (NO), peroxynitrite (cytotoxic to T cells) and by inducing Treg cells and TGF-β secretion [147–149]. By focusing on how the interplays between cancer cells and immune cells influence the redox status in the TME, we will highlight the therapeutic potential of the rational combination of mtROS-modulating agents with cancer immunotherapies.

### Hypoxia-induced mitochondrial ROS stress in the TME

Hypoxia is a prominent feature of the TME of solid tumor and is considered a major factor driving adaptation toward host immunosurveillance evasion. The key molecular mechanism by which cells adapt to hypoxia is through transcription factor, HIF [150]. HIF transcriptionally activates a wide repertoire of genes that promote tumor growth and metastasis. HIF-1 is particularly crucial for shifting the metabolic program of cancer cells from oxidative phosphorylation to glycolysis. Hypoxic tumor, in contrast to non-malignant tissue, to support their energy demands depends more on the anaerobic glycolysis where the final product pyruvate metabolized to lactate to restrict the OXPHOS activity [151].

Contrarily, Gisbergen et al. showed that decrease in OXPHOS results in reduced stabilization of HIF-1α and its downstream targets including carbonic anhydrase IX (CAIX], VEGF [152]. Similarly, there are lot of hypoxic factors influences mitochondria stress phenotype activation and contributes to cancer adaptation and resistance. Developing evidences indicate that ROS produce oxidative stress and regulates immune response for tumor development. [153]. Hypoxia induces production of mtROS through the mitochondrial complex I dysfunction in the inner mitochondrial membrane and the concomitant activation of the mitochondrial Na + /Ca2 + exchanger, NCLX [154]. Hypoxia stabilizes HIF-1α by forming a dimer with HIF-1β which are supported by mtROS production triggering hypoxia-responsive genes to increase angiogenesis [123]. On the other hand, the existence of mtDNA was found to be a mediator of HIF1α and DRP1 relationship under hypoxia in eliciting metabolic reprogramming, and mitochondrial biogenesis in neuroblastoma cells additionally influenced by ROS generated in cytosol [155]. Similarly, in transitional cell carcinoma (TCC), mtDNA amplification under hypoxia alleviates cisplatin induced mitochondrial oxidative stress damage to mtDNA by lowering the mitochondria ROS level and improved the mitochondrial ultra-structural changes resulted in cisplatin resistance [156]. Interestingly, the serum of rectal cancer patients also showed the lower ratio of ROS (reflecting hypoxic tumor) to high cell-free mtDNA damage contributes to systemic inflammation and poor histologic tumor response to neoadjuvant radiotherapy [157]. The intracellular/extracellular transport of the mtDNA is reportedly possible through the extracellular vesicles (EVs) [158] which helps the mtDNA to trigger pro-inflammatory cytokines leading to its own degradation [159]. mtDNA release into cytosol upon elevated ROS also reported to trigger T cell inhibitory function for cancer progression. Hypoxia triggers abundant EV secretions and hypoxia tumor cells derived exosomes contains many mitochondria derived immunosuppressive components and chemokines contributing to tumor progression through macrophage differentiation [160].

### The impact of mitochondrial ROS on T cell, macrophage, and dendritic cell (DC) in the TME

Excessive ROS production leads to chronic inflammation, which is one of the environmental factors that help tumor immunosuppression [161]. Antagonism between immune cells and ROS requires tightly controlled feedback mechanisms to avoid excessive ROS formation [162]. For instance, the ROS levels in NK and T cells need to be delicately controlled to avoid the hazardous effects of high levels of ROS. Yang et al. report that NK cells primed by IL-15 acquire resistance against oxidative stress through the thioredoxin system, and have benefit in protecting other lymphocytes within the TME [163]. It has been well-studied that mild ROS levels are required for proper T cell activation and differentiation, but high and excessive level of ROS upregulates Fas expression and downregulates anti-apoptotic Bcl2 expression to promote T cell apoptosis [164]. On the other hand, proper levels of ROS are needed for the function of antigen-presenting cells. It has been reported extracellular ROS alter the immunogenicity of antigenic peptides, altering T cell priming [165]. Immunogenic cell death (ICD) leads to exhibition and secretion of damage-associated molecular patens (DAMPs), including adenosine triphosphate (ATP), ER protein calreticulin, and nuclear heat-shock protein high mobility group box 1 (HMGB1). These DAMPs interact with their receptors on DCs, leading to DC activation and ultimately antitumor T cell responses [166, 167]. A study shows that scavenging of extracellular ROS using tumor ECM-targeted nanomaterials preserves the stimulatory activity of HMGB1 and restores ICD-induced antitumor immunity [168]. Furthermore, neutrophils, macrophages and MDSCs are known to produce high amounts of ROS to kill tumor [169, 170]. These findings suggest that the level and duration of ROS determine whether ICD occurs and leads to effective antitumor immunity [171, 172]. This is further supported by a study demonstrating that GSH-deficiency in Tregs leads to increased serine metabolism, mTOR activation, and proliferation, resulting in diminished Treg suppressive function in vitro and in vivo [173].

Excluding cancer cells, macrophages are the most immune cells population circulating in the TME that maintain immune homeostasis. In TME, cancer cells remodel the peripheral and distant microenvironment by secreting tumor-derived factors which can stimulate local and circulating monocytes and macrophages to activate and accelerate tumor progression. Macrophages are stimulated by the cytokines secreted by cancer cells, which polarize macrophages with different functions [174]. Macrophages are broadly divided into two categories: classical M1 and alternative M2 macrophages [175–177]. ROS are involved in pro- and anti-inflammatory macrophage phenotypes by contextual fashion [178]. ROS induce some macrophage programming signaling pathway to affect macrophage polarization [179]. For example, M1 macrophage through Nox2 produces ROS to activate NF-κB stimulating phagocytosis [180], but the high level of ROS was harmful to macrophage [181]. The macrophages exhibit similar functions as M2 macrophages that secrete many cytokines, chemokines, and proteases to promote tumor growth, metastasis, angiogenesis, and immunosuppression and they are commonly termed tumor-associated macrophages (TAMs) [182–184]. ROS-induced signaling pathways that promote inflammatory factors secretion in macrophages are well-validated, but little literature mentions the role of mitochondrial chaperone in mtROS induction and macrophages. A recent study showed that mitochondrial Lon is upregulated in differentiation macrophages compared with monocytes and preferential higher in M2-like differentiation macrophages both in vitro and in vivo [81]. This result provides evidence that ROS-induced by Lon in macrophage may play an important role in TAMs differentiation. These results indicated several signaling pathways response to mitochondrial ROS-induced by Lon to promote inflammatory factors present in the TME and contribute to M2d macrophage (TAMs) polarization [185, 186]. For a long time, ROS have been considered as harmful metabolites of mitochondria [187]. More recently, evidence has shown that mtROS are signaling molecules necessary to prevent excessive immune responses, and this concept has also been extended to immunity, in particular to the function of macrophages in which cells predominate [188].

Another group of immune cells that initiate and control immune responses are DCs that can be differentiated from monocytes during inflammation [189]. DCs are crucial for eliciting anti-tumor immunity, due to their ability to present antigens and activate T cells. This antigen-identification process is done by pathogen-recognition receptors (PRRs), like toll-like receptor (TLRs). Starting from immature DCs (iDCs), acceptance of different stimuli guides iDCs to two different physiological types. Once accept proinflammatory cytokines or TLR ligands, LPS, IL-6, would lead to CD80, CD86, and IL-6 expressing mature DCs that drive effector T cell response [190]. While receiving regulatory factors, IL-10, TGF-β, vitamin D3, and corticosteroids, iDCs would become tolerogenic DCs, so-called regulatory DCs (regDCs) that express IL-10, indoleamine 2,3-dioxygenase (IDO), and PD-L1 then dampen effector T cell differentiation or activate Tregs [190]. The differentiation of regDCs and myeloid-derived suppressor cells (MDSC) by TAM promotes the immunosuppressive TME [191–193]. TGFβ and IL-10 secreted by cancer cells and TAMs also inhibit antigen presentation and adaptive immune response promoted by DCs [194–196]. Accumulating evidences have shown that ROS around the TME promote cytotoxicity or immunosuppressive effect of immune cells [197–199], and this issue is based on quantity of ROS in the TME [200, 201]. Moreover, prolong exposed under ROS microenvironment is thought to lead to a chronic inflammatory condition [198]. Diverse inflammatory environments decide antigens cross-presentation capability among DCs and T cells [202]. As a crucial signaling factor in the TME, different levels of ROS may provide some perspectives to elucidate the DC activation state. Various tension of ROS stress levels may result in different terminal outcomes of DC maturation status [202, 203]. Both DCs and T cells showed elevation of intracellular ROS during antigen presentation. This DC-T cell communication was thus inhibited by ebselen, a selenium-containing antioxidant [204]. However, in the dark side of the moon, some cases found that elevated levels of ROS hamper DC cross-presentation and following pro-inflammatory functions. One study showed that mtROS elevation in aged murine DCs obstructed later step of antigen presentation to T cell, this disruption of DC was mitigated after treating with ROS scavenger [205].

### Mitochondrial ROS-induced mtDNA leakage/EV contributes to inflammation and PD-L1-mediated immunoescape

Although our body has strategies to inhibit or kill cancer cells, cancer cells have developed several ways to escape the killing. First, low expression of major histocompatibility complex (MHC) molecule of cancer cells makes cancer cells escape recognition from the immune system. Second, cancer cells gain the stress phenotypes and try to survive under hypoxic and highly oxidative stress of the TME in which immune surveillance will be suppressed. Third, suppression of immune surveillance by releasing inhibitory cytokines (e.g. TGF-β) into the TME and by inhibiting metabolic energy supply. Fourth, activate the immune checkpoint to inhibit the activity of T cells. Immune checkpoints are regulators of the immune system, which are crucial for self-tolerance and prevent the immune system from attacking cells indiscriminately. However, cancers are able to protect themselves from attack by stimulating the immune system.

As signal mediators, ROS also serve a key role in the immune monitoring of regulatory (Tregs) and effector T cells, which rely on toll-like receptors, and perception of the metabolic microenvironment [206]. Prolonged ROS production is considered to lead to chronic inflammation, and inflammatory cytokines and signaling pathways, such as NF-κB and TGF-β, are induced to cause cancer formation and progression [207]. Numerous reports indicated that ROS stress enhances DAMPs production, and mtDNA is pivotal for mitochondrial DAMPs. Due to the bacterial origin of mtDNA which can stimulate innate immune systems including TLR9, NLR family pyrin domain containing 3 (NLRP3), and cGAS-STING signaling pathways in the mammalian cells [158, 208]. ROS promote the mtDNA leakage from mitochondria through more than one mechanism including (1) Bax/Bak pores, (2) VDAC oligomers, (3) mitochondria permeability transition, (4) altered mitophagy, (5) mitochondrial dynamics, and (6) extracellular vesicles [209]. These suggest the extracellular/intracellular release of damaged mtDNA has some physiological role in response to tumor-induced mitochondrial stress. Bao D et al. have established the significance of cytosolic mtDNA stress in cancer progression after DRP1-induced mitochondrial dysfunction leading to tumor-associated macrophage infiltration through HCC secretion of CCL2 by TLR9-mediated NFκB signaling [210]. Their following recent work reported the activation of cGAS-STING signaling contributing to cytosolic mtDNA stress-induced autophagy in esophageal squamous cell carcinoma (ESCC) [211]. Previous evidence also show that oxidative stress can promote the damaged mtDNA escape to the cytosol to upregulate the expression interferon-stimulated genes (ISGs) and activate the interferon (IFN) signal pathway [131, 212–214]. The type II IFNγ is a pleiotropic cytokine with numerous effects on the innate and adaptive immune systems due to the broad expression of its receptors on immune cells [215]. IFN-γ augments the cytotoxic function of NK cells and CTLs to inhibit carcinogenesis [216]. On the contrary, IFN-γ induces many genes involved in cancer cell immunoevasion, such as PD-L1 and CTLA-4, stimulating immune-suppressive mechanisms [217, 218]. Cheng et al. discovered that mitochondrial ROS can promote the immunosuppression gene such as PD-L1 and IDO expression through the STING-IFN axis in various cancers [131]. It has also been reported that ROS-induced PD-L1 expression in macrophages and PD-L1 blockade revert this effect and synergizes with paclitaxel to diminish breast cancer [219]. In addition, mtDNA is vulnerable to damage by ROS and the mtDNA mutations play a role in chemotherapy resistance [220, 221]. The cisplatin resistant cancer cells showed mtDNA mutations and elevated ROS thereby activating NF-κB mediated inhibitor of apoptotic proteins and Ca^2+^-dependent inflammation [84, 222].

Notably, ROS stress can induce the secretion of extracellular vehicles (EVs), which carry mtDNA and PD-L1 to remodel the environment around cancer tissues [131, 223]. ROS-induced EVs further enhance the production of IFN and IL-6 from macrophages, which attenuates T-cell immunity in the TME (Fig. 1). Recent reports indicate that patients with various cancers have an increased level of exosomal PD-L1 which positively correlates with mtDNA and IFN-γ production [131, 224]. The constitute secretion of mtDNA and proteins into EVs is the important phenomenon mutually developed between the cells. The transported materials triggered many cellular events including inflammatory responses favoring or against the pathophysiological process. In the last decade, the extrusion of mitochondrial components to the EVs was reported through the newly included mitochondrial quality control (MQC) pathway called mitochondria-derived vesicles (MDV) which significantly contributes to the organelle homeostasis depending on the severity of dysfunction in mitochondria [225]. The MQC systems help to recover the vital functions of mitochondria. The mechanism of mitochondria-lysosome contact was recently included and considered as one among the MQC involved in cross-talk to deliver components into EVs [226]. MDVs are generated depending on the cargo molecules including proteins and nucleic acids which are limited to one or include cargos from many different compartments of mitochondria [227, 228]. It is more reasonable to consider the fact of MDVs role in eliciting an immune response by allowing oxidized mtDNA to enter the endo-lysosomal pathway and secreted to the extracellular space through exosomes and triggering many inflammatory and anti-inflammatory regulatory pathways [229]. In cancer, although many reported EVs with mtDNA are a critical component affecting the metabolic output and progress the tumor growth, the actual mechanism in MDVs governing the mtDNA transport to EVs is still uncertain. Overall, the MDV dependent MQC mechanisms are important for the cell in achieving both survival and inflammatory properties. More investigations on the biogenesis pathways of MDVs will open a new platform to understand the selection of cargo uptake and its contribution over mitochondrial homeostasis regulation.

## The translational significance of mitochondrial ROS modulation in the prognosis and combination of cancer immunotherapy

In general, the level of ROS in cancer cells is typically higher than their normal surrounding cells. Redox homeostasis in cancer cells can be disrupted by enhancing ROS production or reducing ROS scavenging by inhibiting the antioxidant system. Here, we will focus on the translational and clinical significance of ROS modulation that combines chemo/radiotherapy and immunotherapy against the survival strategies of cancer cells (Table 2).

**Table 2:** The translational and clinical significance of ROS modulation that combines cancer immunotherapy

Treatments that affect ROS modulation	Combination with immunotherapy	Type of cancer	Molecular targeting	Clinical trial/study project and status	Number of participants	Project status and references
Oncoxin-Viusid [OV) -75 ml/day suppresses ROS production	Docetaxel + OV Docetaxel + Radiotherapy + OV	Prostate cancer	Targeting ROS	NCT03543670/Phase 2	25	Completed [303]
Cholecalciferol (Vitamin D3)	Bevacizumab + chemotherapy (Oxaliplatin, Leucovorin Calcium, Fluorouracil, Irinotecan Hydrochloride, Irinotecan) + high-dose vitamin D3	Advanced or Metastatic Colorectal Cancer (SOLARIS)	Vascular endothelial growth factor (VEGF) inhibition	NCT04094688/Phase 3	400	Ongoing
Avastin/Sunitinib	Atezolizumab (MPDL3280A) + Avastin Atezolizumab (MPDL3280A) + Sunitinib	Untreated Advanced Renal Cell Carcinoma	Vascular endothelial growth factor (VEGF) inhibition, Receptor tyrosine kinase inhibitor with PD-1 inhibition	NCT02420821/Phase 3	915	Completed [304]
PARP inhibition suppresses mtROS production	Niraparib + Dostarlimab	Triple negative Breast cancer	PARP inhibition, PD-1 inhibition	NCT04837209/Phase 2	32	Ongoing
Radiotherapy Chemotherapy	Thoracic radiotherapy 30 Gy/10 fractions carboplatin/etoposide + Durvalumab	Extensive Stage Small-cell Lung Cancer (TRIPLEX)	Topoisomerase II Inhibitors, intra- and inter-strand cross-linkage of DNA molecules within the cell, PD-1 inhibition	NCT05223647/Phase 3	302	Ongoing
Radiotherapy Chemotherapy	Neoadjuvant radiotherapy (PTV 41.4 Gy in 23 Fractions) + Paclitaxel (100 mg/m^2^) and Cisplatin (75 mg/m^2^) for 5 weeks + Tislelizumab (200 mg per 3 weeks)	Resectable squamous-cell esophageal cancer	Anti-PD-1 therapy	NCT05323890/Phase 2	15	Ongoing
Radiotherapy Chemotherapy	Stereotactic body radiation therapy (SBRT) + carboplatin + paclitaxel (175 mg/m2) + Durvalumab (1500 mg for every 3 weeks)	Synchronous Oligo-metastatic Non-small cell Lung Cancer	Topoisomerase II Inhibitors, antineoplastic activity, anti-Bcl-2, Anti-PD-1 therapy,	NCT03965468/Phase 2	47	Ongoing
Chemotherapy	Chemotherapy (cisplatin or carboplatin plus vinorelbine or pemetrexed) + Radiotherapy ( lung dose < 20 Gy and/or a lung V20 < 35%) + Durvalumab	Large Volume Stage III Non-small cell Lung Cancer	Anti-PD-1 therapy, tumor shrinkage	NCT04765709/Phase 2	65	Ongoing
Radiotherapy Chemotherapy	Cohort B-D & C-FLOT: Surgery/chemotherapy/radiotherapy + Durvalumab •Paclitaxel/Carboplatin + Durvalumab •Oxaliplatin/ 5-fluorouracil (5-FU)/ Leucovorin/ docetaxel + Durvalumab	Oesophageal Cancer	Anti-PD-1 therapy	NCT02735239/Cohort A1 & A2: Phase 1 Cohort B-D & C-FLOT: Phase 2	73	Completed
Radiotherapy	Radiation-Brachytherapy *brancytherapy (dose = 16 Gy delivered in 2 fractions of 8 Gy per fraction) + Pembrolizumab	Metastatic Esophageal Cancer	Anti-PD-1 therapy	NCT02642809/Phase 1	16	Completed
Chemotherapy	Paclitaxel (80–100 mg/m^2^)/ docetaxel (75 mg/m^2^/ irinotecan 180 mg/m^2^) + Pembrolizumab (200 mg)	Esophageal/ Esophagogastric Junction carcinoma	Anti-PD-1/PD-L1 therapy in patients with PD-L1 CPS ≥ 10	NCT02564263/Phase 3	628	Completed [305] [306]
Radiotherapy Chemotherapy	Cisplatin and Radiation therapy (30 fractions of 60 Gy in 2 Gy) + Pembrolizumab	Head and neck squamous cell carcinomas (HNSCCs)	Improve locoregional recurrence and distant metastatic rates in high-risk patients	NCT02296684/Phase 2	67	Completed [307]
PARP inhibition suppresses mtROS production	Niraparib + Pembrolizumab Niraparib + Dostarlimab	Non-small cell Lung Cancer	PARP inhibition, PD-1 inhibition	NCT03308942/Stage 1/Stage 2	53	Completed

ROS-induced mutational mtDNA, one of the important cellular stresses, can directly regulate the delivery of signal components into the cytoplasm, resulting in mitochondrial retrograde signaling pathways that affect the nuclear gene expression and mitochondrial metabolites to cellular injury. Exosomes, one type of EVs and ranging in size from 30 to 150 nm, act as a medium of cell-to-cell communication to deliver the cargo, including RNA, DNA, proteins, lipids, mitochondria, and mtDNA, to the receptor cells. [229–231]. The secretion of exosomes or EVs indirectly changes the mitochondrial function through the uptake of cargo by the receptor cells such as tumor cells or immune cells [229–231]. The production and composition of EVs affect the oncologic settings, where their concentration is frequently higher in the blood of cancer patients when compared with healthy control [232]. For example, studies involving several cancers showed that tumor-derived exosomes can induce tumor cell proliferation [131, 233–236]. Due to the difference of carrying molecules from origin tumor cell to the peripheral circulation, increasing studies have been described that EVs are as sources of tumor biomarkers in liquid biopsies [237]. Therefore, it seems like EVs should be used to evaluate mtDNA as a biomarker candidate of mitochondrial DAMP. The purpose of liquid biopsy testing is to achieve personalized treatment by identifying the biomarkers of specific physiological and pathological conditions of patient blood. By analyzing EVs/exosomes, liquid biopsy can be used for early diagnosis and subsequent monitoring of disease through simple biosomal fluid testing [238, 239]. Today, the EVs are one of the most exciting and rapidly evolving areas of cancer research in biological fluids. It is recognized that EVs are involved in cell-to-cell communication and are involved in the development of cancer disease. The functionality of the EV makes it ideal for biomarkers based on liquid biopsy. Since EVs represent a mirror of the tissue-specific physiological and pathological condition [240–242], their cargos, RNA and DNA produced from nuclease degradation, can be used for early diagnosis and subsequent monitoring of disease through simple biosomal fluid testing. Therefore, EVs can help physicians choose the best treatment for each patient at all stages of the tumor disease. Taken together, exosomal PD-L1 and mtDNA can serve as a biomarker candidate for cancer diagnosis, prognosis, and cancer immunity therapeutic response [243].

The various antioxidants have been tested as chemo-preventive agents based on the rationale that ROS scavenging can reduce cancer incidence or delay cancer progression [244]. There are also other studies showing that overexpression or targeted catalase, and delivery of SOD or GSH can inhibit tumor growth [245–247]. An early study also demonstrates that administration of the antioxidant NAC suppresses tumor incidence in mice by inhibiting HIF1a-driven tumor growth [248]. But several large-scale clinical trials of dietary antioxidant supplements such as vitamin A, vitamin E, and beta-carotene failed to demonstrate significant antitumor benefits [249, 250]. In some cases, antioxidant supplements even increase the risk of certain cancers [251, 252]. Possible reasons for the unexpected failure of antioxidant approaches include insufficient tumor-promoting ROS scavenging efficiency in mitochondria, and/or interfering with the antitumor effects of ROS in cancer cells or stromal cells in the TME [253–255]. Treatment with paclitaxel can induce extracellular ROS which cause cytotoxic effects to the bystander cancer cells to lethal damage [256]. Paclitaxel also has cytostatic effects to inhibit angiogenesis [257]. A strain of Salmonella typhimurium (VNP20009) has been shown to target and replicate in hypoxia and necrotic areas within tumors with anti-tumor activity in different tumor models [258, 259]. Later, when VNP20009 synergic combined with an anti-angiogenesis inhibitor, endostatin which has no significant anti-tumor effect alone, this strategy significantly enhances therapeutic effects on tumor progressions and normalizes vessels [260].

Numerous studies have demonstrated that chemotherapeutic agents exert tumor-killing effects by generating free radicals that cause irreversible cell damage [261, 262]. Cisplatin, a widely used platinum-based chemotherapy, is known to induce tumor cell apoptosis involving the induction of superoxide but is largely DNA damage-independent, an effect that can be abolished by superoxide scavengers [263]. 5-Fluorouracil, an antimetabolite that interferes with DNA synthesis for the treatment of colon cancer, head and neck cancer, and other solid tumors, induces tumor cell apoptosis by inducing mtROS, and this effect can be inhibited by mitoQ, serves as a mitochondrial-selective antioxidant. [264]. Some chemotherapeutic agents such as taxanes (paclitaxel and docetaxel) and vinca alkaloids (vinblastine and vinblastine) induce the production of superoxide radicals and induce cell death [265, 266]. In addition to chemotherapeutic drugs, ionizing radiation can trigger tumor cell apoptosis through ROS induction and release of mitochondrial cytochrome c [267, 268]. Recently, some new prodrugs have been developed as DNA cross-linkers or ROS-activated alkylating agents. For example, leinamycin (LNM) is a potent antitumor antibiotic produced by *Streptomyces atroolivaceus* S-140. LNM E1 can be activated by cellular ROS oxidation to generate an intermediate with DNA alkylation activity, which exhibits strong cytotoxicity against prostate cancer cell lines with elevated ROS levels [269]. However, Wang et al. reported that fibroblasts facilitate platinum-resistance in ovarian cancer cells by modulating ROS in the TME [270].

In the chronic inflammation TME, tumor cells would balance the lethal level of ROS through regulating the several protective signaling pathways described above as survival strategies. When suffering from hypoxia and nutrient-deprived conditions, the tumor and surrounding stromal cells and endothelial cells begin to secrete pro-angiogenic factors, such as VEGF, angiopoietin, platelet-derived growth factor (PDGF), transforming growth factor beta (TGF-β), fibroblast growth factor (FGF), and some growth factors promotes angiogenesis [271]. Abnormal tumor vasculature is one of the major mechanisms of signaling imbalance induced by pro- and anti-angiogenic molecules [272]. The blood vessels in the TME are very chaotic, complex, irregular, and leaky, resulting in the inability of intratumor hypoxia to deliver antitumor drugs normally. As angiogenesis is the crucial process of tumor progression, targeting angiogenesis is a desirable anti-tumor therapy. Although anti-angiogenesis therapy of Bevacizumab (Avastin) has achieved great success in different cancer treatments, however, anti-angiogenesis is not efficacious as expected because a lot of patients showed resistance to anti-angiogenic therapy. Many papers have proved that antiangiogenic therapies destroy the tumor vasculature, causing intratumoral hypoxia that will promote tumor recurrence and metastasis [273–278]. This reflection indicated that complete inhibition of tumor angiogenesis may not a perfect therapeutic strategy. Abnormal tumor vasculature affects immune cell infiltration through the synthesis of pro-angiogenic factors VEGF and ANGPT2, and promotes TME-mediated immunosuppression [279]. Excessive VEGF in the TME can promote immunosuppression in several ways, such as: regulating T cells to inhibit CTL function [280], inhibiting DC antigen presentation and maturation hindering T cell activation [281], promoting immunosuppressive cells Treg cells, MDSCs and M2 TAM recruitment and proliferation [282]. In 2005, Jain first raised a postulate an emerging concept that “normalize” the abnormal structure and function of tumor vasculature as the anti-angiogenesis therapy [278]. Conceivably, this strategy may alleviate oxidative and hypoxic stresses in the TME and promote the regular vascular formation, immune cells infiltration, and drug delivery into the tumor. However, vascular normalization monotherapy met several challenges, such as the detailed functional mechanisms, the window of normalization monitoring, and time-consuming initiation with short-lived maintenance [278]. The combination idea of anti-angiogenesis and cytotoxic therapies was first postulated by Teicher with many clinical data supported afterward [283]. Vascular normalization has the potential to promote improved efficacy of immunotherapy, and restoring vascular normalization reduces interstitial fluid pressure and improves tumor perfusion, creating a positive feedback loop that not only increases immune cell infiltration within tumors, but also increases oxygen and supply of nutrients to achieve a good therapeutic effect [284, 285]. Therefore, normalization of tumor blood vessels is one of the approaches to solve cancer immunotherapy.

Since immune checkpoint inhibitors, e.g., anti-PD-1, have response rates of only 10–30% in solid tumors because of the immunosuppressive TME. Manipulating the TME therefore is more beneficial for controlling the progression of tumors and reverse the resistance of immunotherapy. Over the past decade, an increasing number of studies have revealed that regulation of the levels of ROS can exert anti-tumor effects by acting on the TME [81]. The combination of metformin with PD-1 blockade enhanced intratumor T cell activation and proliferation, leading to tumor clearance. This observation suggests that non-responders to PD-1 antibodies may have high mROS and more hypoxic microenvironment, which results in compromised T cell response. A study report that adoptive T-cell therapy (ACT) can significantly altered tumor metabolism, leading to GSH depletion and accompanying accumulation of ROS in tumor cells [286]. Some therapeutic molecules, including chemotherapeutics and anti-PD-L1 antibodies, can be delivered to and released within tumor cells or TME via ROS-responsive prodrugs or nanoparticles, thereby inhibiting tumor cell growth in vitro and in vivo [287–289].

Another burgeoning strategy is nanomaterial which can compose of different drugs and be target-specific delivery. To combine with other therapy, such as immunotherapy, many nanoparticles were designed to modulate the level of extracellular ROS to align the immunosuppressive microenvironment. Deng et al. created a nanoscavenger that can be delivered to the low pH microenvironment and anchor to ECM to release drugs to inhibit extrinsic ROS and enhance immunotherapy [168]. The nitric oxide (NO) releasing particles, NanoNO not only normalizes tumor vessels to reprogramme the immunosuppressive tumor microenvironment but also potentiates anti-cancer therapies [290]. A promising therapeutic strategy by dual-targeting particle targeting mTOR efficiently arrest tumor growth by reducing metabolic stresses, repolarized TAMs, inhibiting angiogenesis, reprogramming immune cells [291]. In short, the most important issue is how to deliver the nanoparticle to the target site. Therefore, the top strategy to design a nanoparticles based combination therapy is required to induce the normalized intratumoral vessels to improve immunosuppressive TME.

## Conclusions and perspectives

Cancer is a disease caused by abnormal cell growth and uncontrolled cell death with an ability to spread to other distant tissues. The point of view of cancer research is evolving from a 'cancer cell-centric' perspective to consider tumor as a network of surrounding cells, called a TME. The TME not only includes tumor cells but also their neighbor cells, including CAFs, vascular cells, and immune cells. With extravagance growth, some of stress phenotypes detected in the TME are genome instability (replicative and mitotic stress), hypoxia (metabolic stress and sustained angiogenesis), and the increasing level of ROS (metabolic and mitochondrial stress). ROS act as a double-edged sword in carcinogenesis, which both support and inhibit malignant behavior and the evolution of cancer. ROS produced either by tumor cells or by the TME cells have very diverse effects depending on their level, location, and regulation.

Mitochondria are the major cellular source of ROS production, and mitochondrial ROS (mtROS) are produced during aerobic respiration or as a byproduct of metabolic enzymes. Mitochondria take important roles in cell survival as they contribute to various cellular functions, including ATP production, apoptosis, calcium signaling, mitophagy, and signaling through mtROS. Here, we focus on the impact of mitochondria and mtROS on cancer and immune cells in the TME with their relevance to cancer immunotherapy. Many studies have identified that mitochondrial Lon-ROS promote abnormal cell proliferation, migration, angiogenesis, resistance towards apoptosis, and inflammation. By mtROS-stimulated angiogenesis, migration, and the secretion of inflammatory cytokines and mtDNA/EVs, cancer cells interact with different components in the TME to escape from the immunosuppressive microenvironment.

Two opposing strategies have been attempted to modulate tumor redox as a way to prevent or treat cancer. One approach is to reduce the pro-tumor effects of ROS by reducing oxidative stress with antioxidants. Another approach is to increase cancer cell death by increasing ROS levels in cancer cells. Although many questions remain unanswered, but we know that the effects of ROS-modulating therapies will vary largely depending on ROS level, location, and stage of cancer progression. High levels of ROS induce cellular damage or even cell death. Low to moderate ROS levels promote cell proliferation, EMT, angiogenesis, and inflammation. The increased ROS from cancer cells and various types of myeloid cells in the TME is a characteristic of chronic inflammation, which is intimately involved in cancer development and progression. ROS in the TME are used by cancer cells, immunosuppressive macrophages, and DCs to create an immune tolerance environment for tumors, dampening the outcome of antitumor immunotherapy.

Although current mainstream of cancer therapies is still surgery, chemotherapy, or targeted therapy, the new concept of cancer therapy is trying to keep the tumor in the "hot" state for immunotherapy and to find the weakness of the non-oncogenic addiction for cancer cell survival, avoiding metastasis and recurrence. Combination therapies of the emerging ROS-modulating strategies and cancer immunotherapy enhanced the antitumor effects. Moreover, with help of vessel normalization will mitigate the excess ROS level and hypoxic resistance, which provides a route to drug delivery and immune cells. In summary, the equilibrium of ROS stress in the TME and the immunosurveillance function will optimize the window to enhance the therapeutic efficacy of immunotherapy for eradication of tumors. Rational combination of ROS-modulating agents and immunotherapy is emerging as a promising strategy of cancer treatment. Further research is needed to provide insights on the role of ROS modulators in an immunosuppressive TME to avoid the immunoescape and further recurrence and progression of cancer.

## Data Availability

Not applicable.

## Abbreviations

ACT: Adoptive T-cell therapy
ATP: Adenosine triphosphate
AMPK: AMP-activated protein kinase
ANGPT2: Angiopoietin 2
BAX: Bcl-2-like protein 4
Bcl-2: B-cell lymphoma 2
BNIP3: BCL2/adenovirus E1B 19 kDa protein-interacting protein 3
CAFs: Cancer-associated fibroblasts
CCCP: Carbonyl cyanide m-chlorophenyl hydrazone
CD73: Cluster of Differentiation 73
cGAS: Cyclic GMP-AMP Synthase
CTL: Cytotoxic T lymphocytes
CTLA-4: Cytotoxic T-lymphocyte-associated antigen 4
CuZnSOD: Copper/Zinc superoxide dismutase
DAMPs: Damage-associated molecular patterns
DC: Dendritic cell
DRP1: Dynamin-related protein 1
ECM: Extracellular matrix
EMT: Endothelial mesenchymal transition
ER: Endoplasmic reticulum
ERK: Extracellular signal-regulated kinases
ESCC: Esophageal squamous cell carcinoma
ETC: Electron transport chain
EVs: Extracellular vesicles
FGF: Fibroblast growth factor
FUNC1: FUN14 domain-containing 1
GPx: Glutathione peroxidase
GRP-75: 75-KDa Glucose-regulated protein
GSH: Glutathione
HIF-1: Hypoxia-inducible factor-1
HMGB1: High mobility group box 1
HEY1: Hairy/enhancer-of-split related with YRPW motif protein 1
HMGB1: High mobility group box 1
ICD: Immunogenic cell death
iDCs: Immature dendritic cells
IFN: Interferon
IL-6: Interleukin-6
ISGs: Interferon-stimulated genes
IRF: Interferon regulatory factors
JNK: C-Jun N-terminal kinase
KLF4: Kruppel-like factor 4
H_2_O_2_: Hydrogen peroxide
HIF: Hypoxia-inducible factor
LNM: Leinamycin
MAM: Mitochondria-associated membranes
MAPK: Mitogen-activated protein kinase
MCU: Mitochondrial calcium uniporter
MCL-1: Myeloid cell leukemia-1
MCP-1: Monocyte chemoattractant protein-1
MDSCs: Myeloid-derived suppressor cells
MDV: Mitochondria-derived vesicles
MHC: Major histocompatibility complex
mtDNA: Mitochondrial DNA
mtHSP70: Mitochondrial heat shock protein 70
mtPQC: Mitochondrial protein quality control
mtROS: Mitochondrial ROS
MnSOD: Manganese superoxide dismutase
MQC: Mitochondrial quality control
NAC: N-acetyl-L-cysteine
NAD +: Nicotinamide adenine dinucleotide
NADPH: Nicotinamide-adenine dinucleotide phosphate
NCLX: Mitochondrial sodium/calcium exchanger
NDFUS8: NADH Ubiquinone Oxidoreductase Core Subunit S8
NOX: NADPH oxidase
NF-κB: Nuclear factor kappa-light-chain-enhancer of activated B cells
O2•–: Superoxide
•OH: Hydroxyl radical
8-OHdG: 8-Hydroxydeoxyguanosine
OTC∆: Mitochondrial misfolded ornithine transcarbamylase
OXPHOS: Oxidative phosphorylation
PD-L1: Programmed death-ligand 1
PDGF: Platelet-derived growth factor
PDPN: Podoplanin
PI3K: Phosphoinositide 3-kinase
PINK1: PTEN-induced kinase 1
PRRs: Pathogen-recognition receptors
Prx: Peroxiredoxins
PYCR1: Pyrroline-5-carboxylate reductase 1
PYK2: Proline-rich tyrosine kinase 2
regDCs: Regulatory dendritic cells
ROS: Reactive oxygen species
RT: Radiotherapy
RYR: Ryanodine receptor
SERCA: Sarco/endoplasmic reticulum Ca+ 2–ATPase
SIRT3: Sirtuin 3
SOCE: Store operated calcium entry
SOD1/2: Super oxide dismutase 1/2
STAT3: Signal transducer and activator of transcription 3
STIM1: Stromal interaction molecule 1
STING: Stimulator of Interferon Genes
TAMs: Tumor-associated macrophages
TGF-β: Transforming growth factor beta
TLR9: Toll-like receptor 9
TME: Tumor microenvironment
Tregs: Regulatory T cells
TRP: Transient receptor potential channel
TRPA1: Transient receptor potential cation channel subfamily A member 1
UPRmt: Mitochondrial unfolded protein response
VDAC: Voltage dependent anion channel
VDCC: Voltage-gated calcium channel
VEGF: Vascular endothelial growth factor
YME1L1: ATP-dependent zinc metalloprotease
